# Biologically meaningful regulatory logic enhances the convergence rate in Boolean networks and bushiness of their state transition graph

**DOI:** 10.1093/bib/bbae150

**Published:** 2024-04-05

**Authors:** Priyotosh Sil, Ajay Subbaroyan, Saumitra Kulkarni, Olivier C Martin, Areejit Samal

**Affiliations:** The Institute of Mathematical Sciences (IMSc), Chennai, 600113, India; Homi Bhabha National Institute (HBNI), Mumbai, 400094, India; The Institute of Mathematical Sciences (IMSc), Chennai, 600113, India; Homi Bhabha National Institute (HBNI), Mumbai, 400094, India; The Institute of Mathematical Sciences (IMSc), Chennai, 600113, India; Université Paris-Saclay, CNRS, INRAE, Univ Evry, Institute of Plant Sciences Paris-Saclay (IPS2), 91405, Orsay, France; Université de Paris, CNRS, INRAE, Institute of Plant Sciences Paris-Saclay (IPS2), 91405, Orsay, France; The Institute of Mathematical Sciences (IMSc), Chennai, 600113, India; Homi Bhabha National Institute (HBNI), Mumbai, 400094, India

**Keywords:** cellular automata, gene regulatory networks, network sensitivity, garden-of-Eden (*GoE*) states, *G*-density, *Z*-parameter

## Abstract

Boolean models of gene regulatory networks (GRNs) have gained widespread traction as they can easily recapitulate cellular phenotypes via their attractor states. Their overall dynamics are embodied in a *state transition graph* (STG). Indeed, two Boolean networks (BNs) with the same network structure and attractors can have drastically different STGs depending on the type of Boolean functions (BFs) employed. Our objective here is to systematically delineate the effects of different classes of BFs on the structural features of the STG of reconstructed Boolean GRNs while keeping network structure and biological attractors fixed, and explore the characteristics of BFs that drive those features. Using $10$ reconstructed Boolean GRNs, we generate ensembles that differ in BFs and compute from their STGs the dynamics’ rate of contraction or ‘bushiness’ and rate of ‘convergence’, quantified with measures inspired from cellular automata (CA) that are based on the garden-of-Eden (*GoE*) states. We find that *biologically meaningful* BFs lead to higher STG ‘bushiness’ and ‘convergence’ than random ones. Obtaining such ‘global’ measures gets computationally expensive with larger network sizes, stressing the need for feasible proxies. So we adapt Wuensche’s $Z$-parameter in CA to BFs in BNs and provide four natural variants, which, along with the average sensitivity of BFs computed at the network level, comprise our descriptors of *local* dynamics and we find some of them to be good proxies for bushiness. Finally, we provide an excellent proxy for the ‘convergence’ based on computing transient lengths originating at random states rather than *GoE* states.

## INTRODUCTION

**Figure 1:**
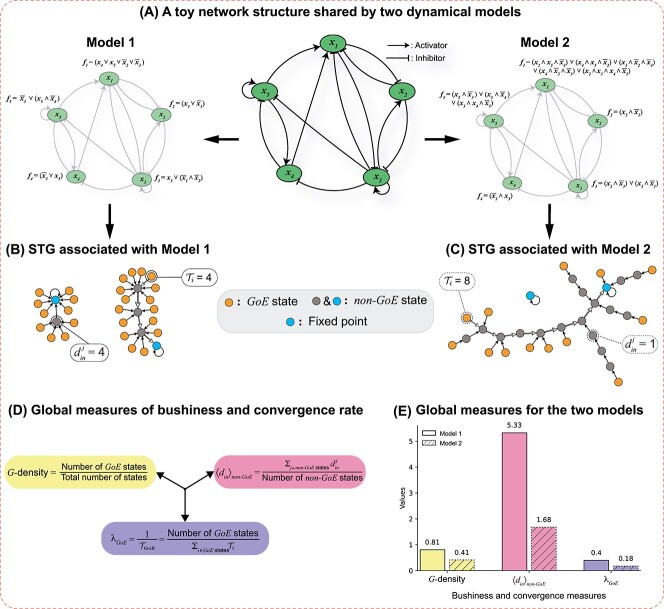
**Measures of ‘bushiness’ and ‘convergence’ of STGs.** (**A**) In the center is a toy network structure with $5$ nodes and $14$ edges. On its two sides are Boolean models (Model 1 and Model 2) with identical network structures but differing regulatory logic rules (as shown at each node). (**B**) and (**C**) display the complete STGs containing $32$ states for both Model $1$ and Model $2$. Visual inspection shows that the STG for Model $1$ appears significantly more bushy than the STG for Model $2$. The transient path from a chosen *GoE* (encircled) to the attractor is traced via the unfilled arrow heads. $\mathcal{T}_{i}$ denotes the transient lengths (steps to reach the attractor) for the $i$th *GoE* state (encircled *GoE* states in the subplot) in the STG. $d_{in}^{j}$ denotes the in-degree of the $j$th *non-GoE* state (encircled grey *non-GoE* states in the subplot) in the STG. (**D**) The global measures that quantify the ‘bushiness’ of the STG are *G*-density and average in-degree of *non-GoE* states ($\langle d_{in} \rangle _{non-GoE}$), whereas the measure that quantifies the ‘convergence’ is the average convergence rate of trajectories originating from *GoE* states ($\lambda _{GoE}$). (**E**) Barplots representing the values of the bushiness and convergence measures described in (D) for Model $1$ and Model $2$. The $x$ and $y$ axes correspond to the three measures and their values, respectively. The bars for the two models for the same quantities have been shown together to illustrate quantitatively the visible differences between (B) and (C).

**Figure 2:**
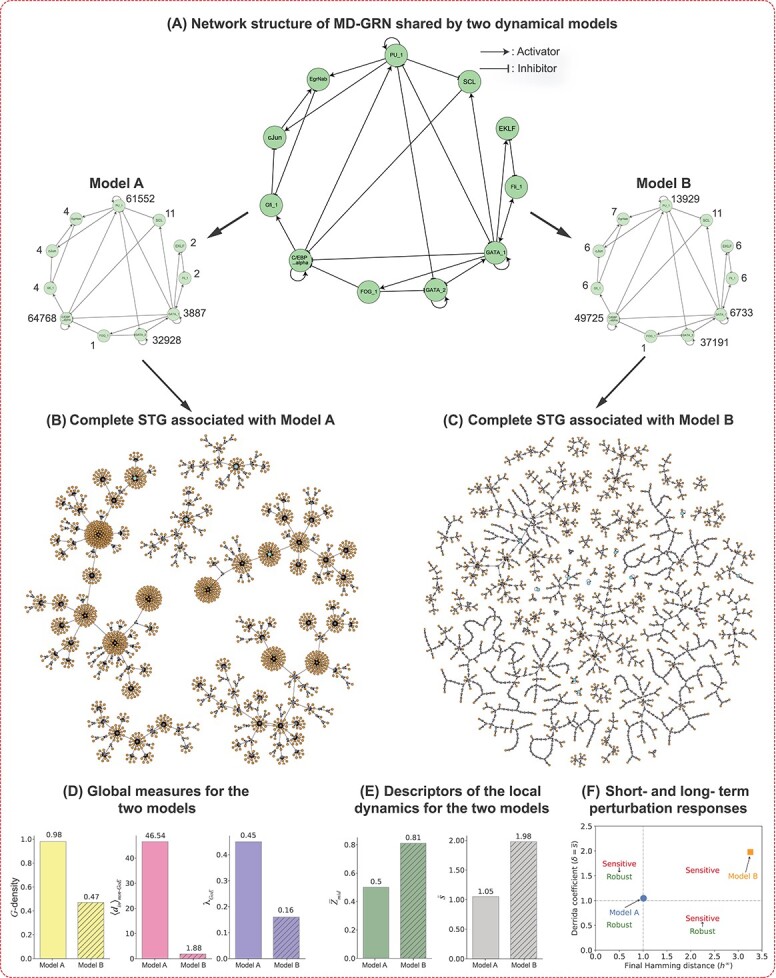
**STGs for two different choices of Boolean rules for the biological network MD-GRN with the values of their global measures of bushiness and convergence, descriptors of local dynamics and short-and long-term perturbation responses.** (**A**) The network in the centre of the top panel is the Myeloid differentiation GRN (MD-GRN). On its two sides are Boolean models (Models A and B) with identical network structure but different regulatory logic rules. For these two models, the genes are arranged in the same manner as depicted in the network structure in the centre. The BFs at each node are encoded as integers as described in supplementary information (SI) text, section 2. In the truth table, the (left to right) order of the inputs for each of the genes in the network is as follows: GATA_2: [GATA_2, GATA_1, FOG_1, PU_1], GATA_1: [GATA_2, GATA_1, Fli_1, PU_1], FOG_1: [GATA_1], EKLF: [GATA_1, Fli_1], Fli1: [GATA_1, EKLF], SCL: [GATA_1, PU_1], C/EBP_alpha: [GATA_1, FOG_1, SCL, C/EBP_alpha], PU_1: [GATA_2, GATA_1, C/EBP_alpha, PU_1], cJun: [Gfi_1, PU_1], EgrNab: [cJun, Gfi_1, PU_1], Gfi_1:[EgrNab, C/EBP_alpha]. (**B**) and (**C**) display the complete STGs containing $2048$ states obtained for Model A and Model B respectively. The ‘orange’, ‘blue’ and ‘grey’ nodes indicate the *GoE* states, fixed points and *non-GoE* states that are not fixed points, respectively. Visual inspection shows that the STG for Model A appears significantly more bushy than the STG for Model B. (**D**) Barplots representing the bushiness (*G*-density, $\langle d_{in} \rangle _{non-GoE}$) and convergence ($\lambda _{GoE}$) measures for Model A and Model B. Each measure is shown as a separate bar plot that quantifies the visible difference between (B) and (C). (**E**) Barplots representing the values of the descriptors of local dynamics ($\overline{Z}_{mid}$ and $\overline{s}$) for Model A and Model B. (**F**) Short ($\delta $)- and long ($h^{\infty }$)- term perturbation responses for the two models. In the ‘Robust’ regime (lower left quadrant), both short- and long-term responses [34] remain below 1, whereas, in the ‘Sensitive’ regime (upper right quadrant), both the responses are above 1; these regimes correspond to ‘ordered’ and ‘chaotic’ dynamics, respectively. The two off-diagonal quadrants indicate disagreement in the short- and long-term responses, an unlikely situation, with the small arrows indicating the change when going from short to long times. Model A lies close to the critical boundary but, Model B lies completely in the Sensitive or disordered regime. Note that $\delta $ is identical to the network sensitivity $\overline{s}$.

For several decades now, complex systems tools have been the cornerstone of quantitative analyses of biological systems, in particular for understanding principles of self-organization [1–8]. Some associated foundations had previously been laid in the field of cellular automata (CA) in attempts to apply automaton and computational theories to living systems [9–12]. Till date, a wide range of automata have been studied such as 2D CA [9], 1D elementary CA [10, 13, 14], non-uniform CA [15] and sequential dynamical systems [16] to name a few. Stuart Kauffman proposed that gene networks might be aptly modeled by generalizations of automata, namely via Boolean networks (BNs) [1, 17, 18], in which genes can assume 2 states—‘on’ or ‘off’. Notably in the recent past, several design principles that are specific to reconstructed biological BNs have been identified. Some of these include the preponderance of regulatory logic that minimize complexity [19], the prevalence of redundant pathways in such networks [20], the predominance of network architectures that lead to both minimal frustration of steady states [21] and critical dynamics [22]. BNs have been extensively used to model the dynamics of gene regulatory networks (GRNs) to explain a wide range of biological processes including differentiation, metabolism, apoptosis and proliferation, among many others [23–31]. The full dynamics of a BN can be described by a state transition graph (STG) in which a fixed point attractor typically corresponds to a biological steady-state expression pattern specific to a cell type, while its basin of attraction corresponds to all state expression patterns that will converge to that steady state under the BN dynamics [17, 32, 33] (see Figures 1 and 2). Such dynamics must exhibit robustness to perturbations if cells are to maintain homeostasis [26], making it imperative to investigate features of the STG associated with robustness and how regulatory logic rules can impact those features.

In the framework of elementary CA, Stephen Wolfram systematically classified all CA rules into four classes based on the dynamical behavior they exhibited [35]. To date, an analogous framework that categorizes global dynamical properties of BNs based on types of Boolean function (BF) has not been proposed, though there have been extensive studies on how network structure and BFs can affect the dynamics of BNs [20, 36–39], dynamical regimes in random BNs [24, 40–43] and in reconstructed BNs [19, 22]. Thus, we ask here how different classes of regulatory logical rules in BNs can affect systemic dynamics. For that, we provide mathematical measures that build on previous work studying STGs in CA, especially by Andrew Wuensche [44–46]. We concentrate on characterizing the overall global dynamics based on two features: the rate of contraction—hereafter referred to as ‘bushiness’—and the rate of ‘convergence’. The term ‘bushiness’ serves as a visual metaphor associated with the average number of edges feeding into nodes of the STG. To illustrate that, consider two Boolean models (Model A and Model B) derived from a myeloid differentiation GRN (MD-GRN) [29] that differ in the type of regulatory logic rule used but recover the same biological attractors (see Figure 2(A)). Figures 2(B) and (C) display the complete STGs for Models A and B, respectively. Clearly, the STG of Model A appears highly ‘bushy’ compared to that of Model B. We quantify such bushiness using two measures. The first is given by the reduction of the space of states when applying the update dynamics. In continuous-time dynamical systems, the analog of this reduction is the contraction rate specifying how phase space volume diminishes in time; in reversible dynamics such as in Hamiltonian systems, phase space volume is conserved, while in dissipative systems, phase space gets contracted. In our discrete-time case, we quantify this reduction by comparing the number of states produced from one update of the BN to the initial number of states. It is easy to see that the reduction factor is just the fraction of states that have in-degree equal to 0 in the STG. Such states are referred to as *garden-of-Eden* (*GoE*) states, so we will be analyzing the so-called *G*-density equal to the fraction of states that are *GoE* states. The second is based on the in-degree of nodes, taking the average over all nodes that have incoming edges. Both of these measures quantify the *irreversibility* of the BN dynamics and we shall see that they are closely related. On the other hand, we quantify *convergence* based on the length of trajectories (or, transient lengths) originating at *GoE* states because these give the maximal trajectory lengths. The three quantities, namely (1) *G*-density, (2) average in-degree of *non-GoE* states and (3) average convergence rate of the trajectories originating at *GoE* states, will be referred to as *global* measures throughout this manuscript (see Methods for the definitions and Figure 1 for visual illustrations). In CA, Wuensche observed that highly bushy STGs with shorter transients have more ordered dynamics while less bushy STGs with longer transients have more chaotic dynamics [47]. So far, studies of such structural features of STGs have been primarily restricted to elementary CA and random BNs [14, 44, 48, 49], with no such investigation in reconstructed Boolean GRNs. Here we address this challenge and, in doing so, we will exhibit the strong association between bushiness, rate of convergence and dynamical *robustness* which is an essential feature of biological systems. This last characteristic is exemplified via a Derrida plot (SI Figure S1) [24, 50] showing the system’s response to a perturbation after one time step for the two models A and B. The behaviour at long time can also be considered (see Figure 2(F)) [34]. Furthermore, investigating these properties will enable us to select suitable models [51] among multiple possibilities, helping us identify models that exhibit greater robustness.

Unfortunately, obtaining the STG and its features can be computationally expensive. In particular, all three measures we introduce here require listing the *GoE* states, a task that becomes computationally intractable with increasing network size [52, 53]. It is thus appropriate to search for proxies of those three global measures that remain tractable even for large networks. Although this was not done explicitly by Wuensche, in his studies of CA, he introduced the $Z$-parameter, a quantity that can be computed directly from the rule table that measures the probability that the next unknown cell in a partial pre-image is uniquely determined [45, 54, 55]. He showed that the $Z$-parameter is a useful predictor for the bushiness of the STG [45]. In extending the $Z$-parameter to BNs, one is confronted with the fact that in contrast to the situation in elementary CA, each node in a BN is allowed to have a different ‘rule’ and there is no natural ordering of its inputs, making it non-trivial to adapt the $Z$-parameter to BNs. This challenge of finding rule-based measures in BNs has not been addressed so far, so we will fill that gap.

The primary objectives of this work are to delineate how different classes of logic rules, namely effective functions (EFs), effective and unate functions (EUFs), read-once functions (RoFs) and nested canalyzing functions (NCFs), shape the structural features of the STG of a Boolean GRN, and search whether there exist good proxies for those features. To do so, we first select $10$ published reconstructed Boolean GRNs and for each one generate four ensembles of models, having the same network structure and the same set of biological fixed points but differing in the type of logic rule employed. We consider different *global* measures to characterize two features of the structure of STGs: (1) bushiness and (2) rate of convergence. We determine how these measures vary across the four ensembles for each of the $10$ GRNs, particularly how distinct the effects of using biologically meaningful BFs, i.e. bmBFs (EUFs, RoFs and NCFs) are compared to using EFs (which are random BFs for all practical purposes). Next we explore the correlation between bushiness and convergence. Following that, we address the challenge of adapting the $Z$-parameter to BFs in BNs by proposing a scheme based on the permutation of inputs to a BF. Subsequently, we posit four variants of the $Z$-parameter at the level of the BF and their respective counterparts at the level of the network which we collectively refer to as the network $Z$-parameters. One last quantity we consider is the so-called network sensitivity or average sensitivity of a network [22, 56]. We then study the distributions of these *local* descriptors of dynamics, that is, the four network $Z$-parameters and network sensitivity, in our ensembles and evaluate correlations between those descriptors. Finally, we inquire whether these descriptors of local dynamics, in addition to another measure, can serve as ‘proxies’ for the bushiness and convergence. It is noteworthy to mention that while the descriptor ‘network sensitivity’ has been linked to robustness of BNs, its connection to the structural features of the STG has not been investigated prior to this study as per our knowledge.

In order to facilitate readability, all abbreviations and major symbols used in this manuscript, along with their expanded forms, are provided in SI Table S1.

## METHODS

### Background on Boolean modeling

BNs are comprised of nodes (genes or proteins) and directed edges between nodes, which are associated with the regulation of each target gene by its controlling genes [18]. We denote the state of the BN at time $t$ by the vector $\mathbf{X}(t)$ whose $i^{\text{th}}$ entry ($i^{\text{th}}$ node) is $x_{i}(t)$, where $i \in \{1,2, \ldots , N\}$ ($N$ is the number of nodes in the network) and $x_{i} \in \{0,1\}$. Each node $i$ is assigned a BF (or *logical update rule*) $f_{i}(x_{i}^{1}(t), x_{i}^{2}(t),\ldots , x_{i}^{k}(t))$ that acts on its $k$ inputs $x_{i}^{m}(t)$ ($m \in \{1,2,\ldots ,k\}$) to return its state $x_{i}(t+1)$. The number of input combinations $(x_{i}^{1}(t), x_{i}^{2}(t),\ldots , x_{i}^{k}(t))$ for which $x_{i}(t+1) = 1$ is the bias $P$ of the BF $f_{i}$. Of all possible BFs, some classes are *biologically meaningful* in that they satisfy properties that ‘real’ regulatory logic are expected to possess such as effectiveness, unateness and canalyzation [1, 57, 58]. The bmBFs included in this work are EUFs, RoFs and NCFs. See SI text, sections 1, 2 and 3 for detailed information on BNs, BFs and bmBFs, respectively.

If all nodes are updated simultaneously, the update scheme is said to be *synchronous* [1], otherwise it is *asynchronous* [59]. Under synchronous dynamics, two dynamical outcomes are possible: (a) a *fixed point attractor*, *i.e.*, a state that, on further update, remains unaltered, or (b) a *cyclic attractor* (or *cycle*), in which the network keeps cycling through a fixed set of states indefinitely on further updates. Figures 1 and 2 illustrate BNs and their basins of attraction. Asynchronous updates lead to the same set of fixed points and may be considered more realistic but they are much more difficult to analyze [60] and are hence out of the scope of this study.

### Generating ensembles of biologically plausible models of 10 published Boolean GRNs

This section describes our procedure to generate ensembles of biologically plausible Boolean models. We first choose $10$ reconstructed Boolean GRNs that are published in the literature (their abbreviations and references are provided in Table 1; the models in BoolNet format in SI Tables S2–S11; types of BFs used in SI Tables S12–S21; their biological fixed points in SI Tables S22–S31) and for each GRN, we generate four biologically plausible ensembles, all of which share the same network structure of the GRN and satisfy the same biological fixed points [51, 61], but differ only in the types of BFs used, namely, EFs, EUFs, RoFs and NCFs.

**Table 1:** 10 published reconstructed Boolean GRN models. ‘Names of models’ column provides the names of each of the Boolean GRN network structures that are used in this work. The ‘Abbreviation’ column provides the abbreviated form we designate for each of the $10$ models. The ‘Nodes’ and ‘Edges’ columns provide the number of nodes and edges, respectively, for each of the $10$ models

**S. No.**	**Names of models**	**Abbreviation**	**Nodes**	**Edges**	**Publication year**	**PMID/ others**	**Reference**
1	Root Stem Cell Niche GRN	RSCN-GRN	9	19	2010	$20920363$	[62]
2	Epithelial-Mesenchymal Transition GRN	EMT-GRN	12	40	2023	$36968076$	[63]
3	NeoCortex Developmental GRN	NCD-GRN	10	19	2010	$20862356$	[64]
4	Early Heart Development GRN	EHD-GRN	15	39	2012	$23056457$	[65]
5	Myeloid Differentiation GRN	MD-GRN	11	30	2011	$21853041$	[29]
6	T-Helper Cell differentiation GRN	THC-GRN	23	34	2006	$16542429$	[25]
7	EGFR signalling pathway	EGFR-GRN	13	21	2019	$30582550$	[66]
8	Epithelial-Mesenchymal Transition (with Senescence) GRN	EMT-Senescence-GRN	9	30	2023	Conference Proceeding	[67]
9	Flower Organ Specification GRN	FOS-GRN	13	42	2010	$20303988$	[68]
10	Gonadal Sex Determination GRN	GSD-GRN	19	75	2015	$26573569$	[69]

Our methodology to generate such ensembles using these types of BFs is detailed in the SI text, section 4 [51, 61]. We shall refer to these ensembles (sampled or exhaustively enumerated) of models as EF-ensemble, EUF-ensemble, RoF-ensemble and NCF-ensemble (SI text, section 4; SI Tables S32–S41). Details of the 10 reconstructed GRNs are provided in SI text, section 5. As it was computationally infeasible to generate all the allowed UFs for nodes with more than six inputs, we proposed an algorithm to sample UFs (and EUFs). Our algorithm is based on an iterative color propagation scheme on the vertices of the Boolean hypercube (SI text, section 4) that is induced by the monotonicity of the output as a function of inputs (SI Figures S2 and S3). The distribution of UFs sampled using this algorithm is not perfectly uniform (SI Figure S4); however, it does not deviate much from the uniform distribution.

We generated the STGs for all 21,032,377 models across all $10$ GRNs and all ensembles using the R package BoolNet [70] and compute the global measures described in the next section. Using large ensembles of models ensures the statistical reliability of our results.

### Global measures of bushiness and convergence of the STG

We quantify ‘bushiness’ of the STG by defining two quantities: (a) *G*-density and (b) average in-degree of the *non-GoE* states. ***G*-density** is defined as the fraction of states that are *GoE*. This quantity captures the degree of contraction or irreversibility of the dynamics, measuring the fraction of states removed from phase space after a single update. The average in-degree of *non-GoE* states is defined as the average of the number of predecessors (in-degree) of *non-GoE* states, a quantity we denote by $\boldsymbol{\langle d_{in} \rangle _{non-GoE}}$. Furthermore, we show (SI text, section 6) that $\langle d_{in} \rangle _{non-GoE}$ can be expressed as a function of the *G*-density as follows: 


(1) \begin{eqnarray*} \langle d_{in} \rangle_{non-GoE} = \frac{1}{1-G\text{-density}} \end{eqnarray*}

We quantify the ‘convergence’ of the STGs by defining the *average convergence rate* of trajectories originating at *GoE* states ($\boldsymbol{\lambda _{GoE}}$) as follows: 


(2) \begin{align*}& \lambda_{GoE} = 1/\mathcal{T}_{\scriptscriptstyle GoE}\end{align*}

where $\mathcal{T}_{\scriptscriptstyle GoE}$ is the average of the lengths of transients originating at *GoE* states. All states that are not members of the attractor are called ’transient’ states.

To illustrate how these measures can be computed, we take simple cases shown in Figure 1(A) that are based on toy models (Model $1$ and Model $2$) which share the same network structure but differ in the BFs used. Their associated STGs (Figures 1(B) and (C)) show contrasted levels of bushiness and convergence. Clearly, the three measures computed on the STG (Figure 1(D)) of the toy Model $1$ (Figure 1(B)) are higher than that for the STG of Model $2$ (Figure 1(C)) as provided in the barplots (Figure 1(E)). From these, the reader may expect the bushiness and convergence to go hand in hand but, in fact, the situation is more subtle as we will demonstrate later in the Results section.

As a variant to $\lambda _{GoE}$, we also consider the reciprocal of the average of the transient lengths of trajectories originating at *all* states of the STG, which we denote as $\lambda _{all} (=1/\mathcal{T}_{\scriptscriptstyle all})$. This quantity has the advantage that it can be estimated numerically via sampling, and we denote the associated (stochastic) estimator as $\lambda _{random} (= 1/\mathcal{T}_{\scriptscriptstyle random}$).

### Adaptation of *Z*-parameter in CA to BNs

In CA, the $Z$-parameter as defined by Wuensche reflects the degree to which pre-images of states in the state space are identifiable for a given automaton update rule [54] (SI text, section 7). A low $Z$-value indicates a lower probability to have a unique pre-image, that is, a lower degree of reversibility of the dynamics [14, 45, 46, 54]. A $Z$-parameter value of $1$ corresponds to perfectly reversible dynamics. We may thus expect a lower $Z$ value to be associated with a higher in-degree of states in the STG and thereby a higher *G*-density.

For any one-dimensional CA rule, Wuensche introduced a computational procedure to define $Z_{left}$ and $Z_{right}$, associated with reading the spatial arrangement of inputs from left to right and from right to left respectively, and then he defined the $Z$-parameter as their maximum [45] (SI text, section 8). Since there is no notion of ‘left’ or ‘right’ of a node for BFs in BNs, the above definition needs to be extended.

#### Calculation of *Z*_*left*_

**Figure 3:**
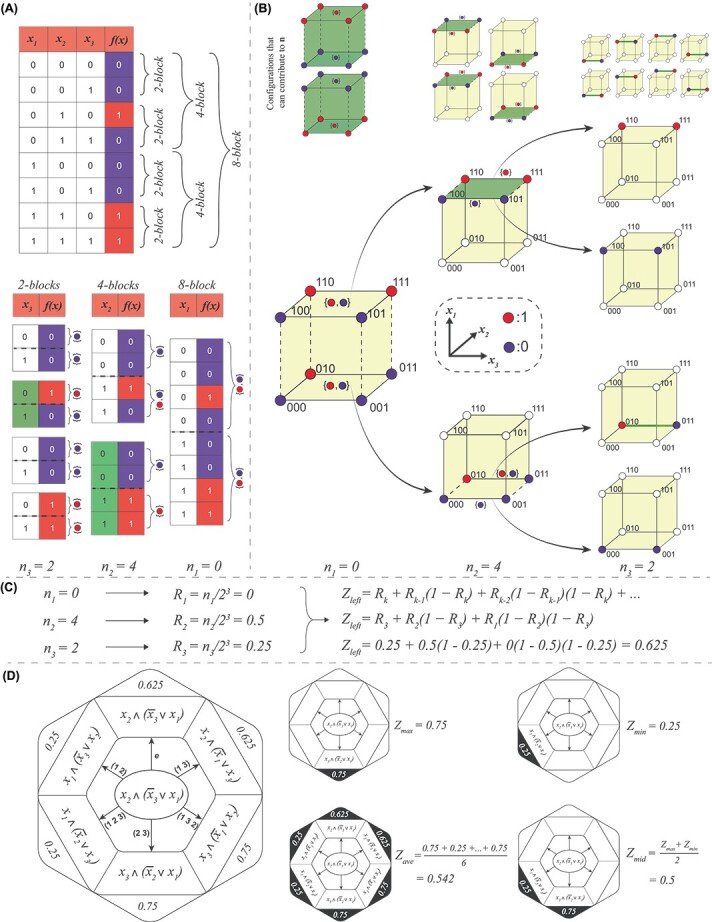
**Calculation of** $\boldsymbol{Z}$ **-parameters of BFs in BN models**. (**A**) Calculation of the vector **n** from the truth table as proposed by Wuensche. Top panel: the truth table of a particular BF for which the $Z$-parameters will be computed. The colors red and blue correspond to the output values $1$ and $0,$ respectively. The list of $2^{i}$-blocks are shown via braces, where $i \in \{1,2,3\}$. Bottom panel: for every $2^{i}$-block, obtain the values spanned by the output in its top (respectively bottom) half (these halves are separated by the dashed lines). The corresponding values are shown to the right of the braces. If there is a single value for both top and bottom braces and if these are different, then we have a CC-$2^{i}$-block and it contributes $2^{i}$ to $\mathbf{n}$. The corresponding cells in the column of the $(k-i+1)$th variable are colored in green. $n_{1}$, $n_{2}$ and $n_{3}$ are the contributions from the CC-$2^{3}$-, CC-$2^{2}$- and CC-$2^{1}$-blocks (of values 0, 4 and 2 here) defining the vector **n**. (**B**) An equivalent formalism for calculating **n** on the Boolean hypercube. This figure illustrates how elements of **n** can be computed by recursively splitting the Boolean hypercube into two hyperplanes, obtaining at each step an element of **n** starting from $n_{1}$. Top panel, from left to right: the cubes show which configurations (vertex colorings) can contribute to $n_{1}$, $n_{2}$ and $n_{3}$, respectively. For instance, a $2^{3}$-block contributes to $n_{1}$ if and only if all the vertices in the plane $x_{1} = 0$ are colored red (or dark blue) *and* all those in plane $x_{1} = 1$ are colored dark blue (or red), as shown at the top left of this sub-figure. Bottom panel: by comparing the actual node coloring to the templates contributing in the top panel, we see that there is no contribution to $\mathbf{n}$ from the $2^{3}$-block, so $n_{1}=0$. The next step of the recursion (just to the right) concerns contributions of $2^{2}$-blocks (associated with two faces of the cube). Here, the upper face (colored dark green) contributes to $n_{2}$, whereas the lower face does not (based again on comparing to the templates just above them). Finally, still on the right of that, we have the case of the $2^{1}$-blocks that may contribute to $n_{3}$. We have colored in green the ones that do contribute. Reaching the $2^{1}$-blocks marks the end of the recursion. Note that if a grouping (cube, face or in higher dimensions any hyperplane) contributes to $\mathbf{n}$, then none of its sub-parts will contribute to $\mathbf{n}$. (**C**) This sub-figure summarizes the computation of $Z_{left}$ of a BF once the vector $\mathbf{n}$ is obtained. (**D**) Computation of the four $Z$-parameter variants: $Z_{max}$, $Z_{min}$, $Z_{ave}$ and $Z_{mid}$. Left panel: the Boolean expression of the BF in the truth table in (A) is $x_{2} \land (\overline{x}_{3} \lor x_{1})$, as displayed in the center of the hexagon. We then apply the $k!$ permutations (cf. arrows) to produce the (distinct) permuted BFs as shown at the end of these arrows. The values shown in the outer hexagon are the $Z_{left}$ values for each of the permuted Boolean expressions. Right panel: a hexagon is produced for each of the four variants of the $Z$-parameter: $Z_{max}$ is the maximum $Z_{left}$ value over all permutations. $Z_{min}$ is the minimum $Z_{left}$ value over all permutations. $Z_{ave}$ is the average of the $Z_{left}$ over all permutations. $Z_{mid}$ is the average of $Z_{max}$ and $Z_{min}$.

For a given ordering of the inputs, we can follow Wuensche’s definition of $Z_{left}$ which is computed from a certain vector $\mathbf{n}$ whose entries are obtained from an iterative scheme to count all the ‘complement canalyzing’ blocks of a truth table, a property which we abbreviate as CC-$2^{i}$-block (see Figure 3(A) and SI text, section 8). Figure 3(B) illustrates our alternate approach to compute $\mathbf{n}$ that is based on recursively visiting $(k-i+1)$ dimensional hyperplanes (these have a direct correspondence with the $2^{k-i+1}$-blocks), going from the largest blocks to the smallest ones and checking at each step of the recursion whether the hyperplanes contribute to $\mathbf{n}$ (see SI text, Algorithm 1 for the associated pseudocode). Figure 3(C) shows the formula to compute $Z_{left}$ using $\mathbf{n}$. Some interesting properties of $Z_{left}$ for any BF include invariance both under the negation of any of the inputs (SI text, section 9, Property 1) and under complementation (SI text, section 9, Property 2).

#### Defining other *Z*-parameters

In this section, we propose a number of extensions to Wuensche’s $Z$-parameter for BFs. Since there is no specific spatial order or arrangement of the inputs to a node in a BN, we define our $Z$-parameters in a manner that accounts for all permutations on its inputs (SI text, section 2). Given a $k$-input BF, we compute the $Z_{left}$ for all $k!$ permutations on its inputs, from which we define four $Z$-parameter variants: $\boldsymbol{Z_{max}}$, $\boldsymbol{Z_{min}}$ and $\boldsymbol{Z_{ave}}$ are the maximum, minimum and average, respectively, and $\boldsymbol{Z_{mid}}$ is the average of $Z_{max}$ and $Z_{min}$ (Figure 3).

The analytical formulas to compute $Z_{max}$, $Z_{min}$ and $Z_{mid}$ for $k$-input NCFs with bias $P$ are (see SI text, section 9 for proofs): 


(3) \begin{align*} Z_{max}^{NCF} &= \left\{\begin{array}{ll} \frac{P}{2^{k-1}} \text{ if } P < 2^{k-1},\\ 2 - \frac{P}{2^{k-1}} \text{ if } P> 2^{k-1} \end{array}\right.\end{align*}

(4) \begin{eqnarray*} Z_{min}^{NCF} &\!\!\!\! = \frac{1}{2^{k-1}}\qquad\qquad\qquad\qquad\end{eqnarray*}

(5) \begin{align*}\ Z_{mid}^{NCF} &\, = \left\{\begin{array}{ll} \frac{1+P}{2^{k}} & \text{if } P < 2^{k-1}\\ 1-\frac{P-1}{2^{k}} & \text{if } P> 2^{k-1} \end{array}\right.\end{align*}

More explicitly, $Z_{min}$ is dependent only on the number of inputs ($k$), while $Z_{max}$ and $Z_{mid}$ vary linearly with the bias $P$ for $0 < P < 2^{k-1}$ and $2^{k-1} < P < 2^{k}$ for a fixed $k$, making their computations efficient and scalable for NCFs.

Regarding $Z_{ave}$, we found that its computation for any $k$-input BF as mentioned above is equivalent to computing the average of $Z_{left}$ values only for the *non-equivalent* permutations of that BF, leading us to two conjectures, both of which we prove in SI text, section 10: (1) the number of non-equivalent permutations divides $k!$ (2) each of the $m$*non-equivalent* permutations occurs an equal number of times. Furthermore, the fraction of non-equivalent permutations of BFs decreases with increasing number of inputs for BFs randomly drawn from NCFs and RoFs, and for BFs in reconstructed BNs, whereas it increases for BFs randomly drawn from EFs or EUFs (SI Figure S5). In sum, these results enable us to significantly reduce the computation time of $Z_{ave}$ in practice.

We define the *network*$Z$-parameter as the average of a considered $Z$-parameter over all the nodes of a BN and denote it by $\overline{Z}_{x}$, where $x \in \{max,min,ave,mid\}$: 


(6) \begin{align*}& \overline{Z}_{x} = \frac{1}{N} \sum_{i=1}^{N}{Z_{x}^{i}}\end{align*}

Here, $i \in \{1,2, 3,\ldots , N\}$ is the index of a node in the BN with $N$ nodes, and ${Z}_{x}^{i}$ is the $Z$-parameter of the BF at node $i$.

### Average sensitivity of a BF and network sensitivity

The average sensitivity of a BF indicates its sensitivity to one-bit flips of its inputs [56]. For a $k$-input BF, the average sensitivity ($s$) is given by 


(7) \begin{align*}& s = \left\langle\ \sum_{i=1}^{k} f(x \oplus e_{i}) \oplus f(x) \right\rangle_{x}\end{align*}

where $\oplus $ is the logical XOR operator and $e_{i} \in \{0,1\}^{k}$ is the unit vector with $x_{i} = 1$ and $x_{j} = 0\ \forall\ j \ne i$. The *network* sensitivity, $\overline{s}$, is the mean, over all nodes of the network, of the average sensitivities of the associated BFs [22, 56].

## RESULTS

Here, we study the robustness of different BNs via their degree of contraction and convergence for models in ensembles that are obtained from 10 reconstructed GRNs (see Methods), and identify which properties of the underlying rules drive high bushiness (contraction) and convergence. Furthermore, we provide a connection between the values of the above-mentioned ‘global’ measures of robustness using the STG and those of descriptors of ‘local’ dynamics. For brevity, the main part of the manuscript presents results only for four of the $10$ reconstructed GRNs (RSCN-GRN, EMT-GRN, NCD-GRN and EHD-GRN), with the remaining six GRNs provided in the SI. Also, in what follows and unless stated otherwise, we use the term ‘distribution’ of a quantity (global or local measures) to mean the distribution of that quantity in any one of the four ensembles.

### Biologically meaningful functions lead to highly bushy and convergent STGs

**Figure 4:**
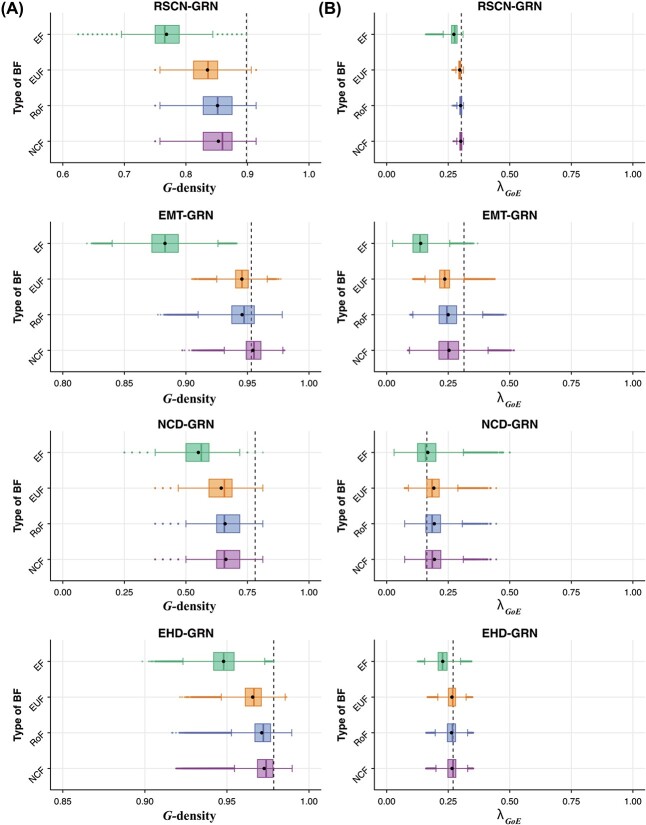
**Distribution of global measure values for different ensembles generated using network structures from four published Boolean GRNs**. The box plots in (**A**) and (**B**) display the distributions of *G*-density and $\lambda _{GoE}$ respectively in the four ensembles that each use a given type of regulatory logic. The mean and median of the distributions are indicated by the black dot and the vertical line within the box respectively. The vertical dashed lines in (A) and (B) correspond to the *G*-density and $\lambda _{GoE}$ respectively for the Boolean model provided by the modelers in the published article.

As outlined in the Introduction section, we utilize a pedagogical example (see Figure 2) of a myeloid differentiation GRN, to elucidate the implication of choice of models on the global dynamics. Here, we would like to unveil the fact that, the functions in Model A are chosen from the biologically meaningful class (specifically, NCF) whereas, the functions in Model B are random EFs. While both of the models yield the same biological attractors, Model A demonstrates more bushy and convergent STG (a signature of robust or ordered dynamics) compared to Model B (Figure 2). To explore the implications of various function classes on the global dynamics more broadly, we examine the distributions of the global measures across different ensembles (see Methods) for the 10 published Boolean GRNs. We first obtain the distribution of the *G*-density as shown by the box plots in Figure 4(A) and SI Figure S6. We observe two striking and consistent patterns across all $10$ GRNs (Figure 4(A) and SI Figure S6). First, STGs in ensembles employing bmBFs (EUF-ensemble, RoF-ensemble and NCF-ensemble) are more bushy than STGs in the EF-ensemble, an indication of more robust dynamics. Second, the STGs in the NCF-ensemble are typically more bushy than those in the EUF-ensemble or RoF-ensemble. Given this trend for robustness, if one performs model selection during Boolean GRN reconstruction, it is appropriate to adhere to biologically meaningful classes [51]. Moreover, to enforce the robustness constraint systematically, one can focus on the models with top *G*-density values within those classes, significantly narrowing down the search space. Similar results follow for the distribution of the average in-degree of *non-GoE* states ($\langle d_{in} \rangle _{non-GoE}$) since it is a non-linear monotone increasing function of *G*-density as derived in Equation (1) (SI Figures S7 and S8).

Next, we consider the distribution of the *average convergence rate* of trajectories originating at *GoE* states ($\lambda _{GoE}$). From Figure 4(B) and SI Figure S9, we observe that on average, the models in the EF-ensemble have lower convergence (smaller values of $\lambda _{GoE}$) in comparison to ensembles that employ bmBFs. We also find that the ensembles constrained with bmBFs have similar values of $\lambda _{GoE}$. These trends are consistent across the $10$ GRNs as can be seen in Figure 4(B) and SI Figure S9.

Lastly, the Spearman correlation coefficients between *G*-density and $\lambda _{GoE}$ for ensembles across the $10$ GRNs (SI Figures S10 and S11) reveal a positive correlation but that is surprisingly small, indicating that the two measures capture different structural characteristics of the STG.

### Biologically meaningful BFs have lower local dynamics descriptor values

**Figure 5:**
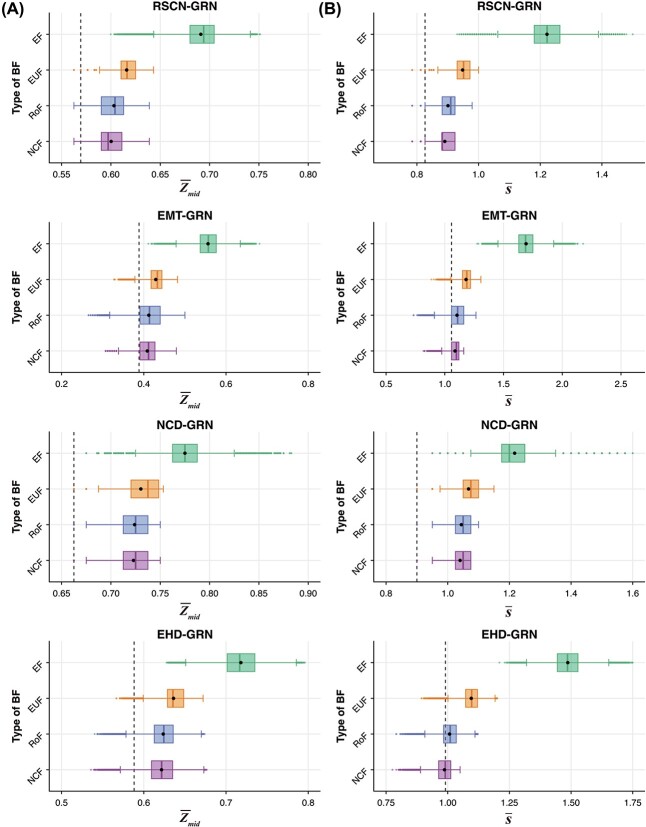
**Distribution of the values of the descriptors of local dynamics for different ensembles generated using network structures from four published Boolean GRNs**. The box plots in (**A**) and (**B**) display the distributions of $\overline{Z}_{mid}$ and $\overline{s}$ respectively in the four ensembles that each use a given type of regulatory logic. The mean and median of the distributions are indicated by the black dot and the vertical line within the box respectively. The vertical dashed lines in (A) and (B) correspond to the $\overline{Z}_{mid}$ and $\overline{s}$ respectively for the Boolean model provided by the modelers in the published article.

To bypass the computational challenges encountered when computing global measures for larger networks, we explore five descriptors of local dynamics (see Methods) as potential proxies. Consequently, we begin by examining their distributions across different ensembles. The distributions of $\overline{Z}_{max}$ (SI Figures S12 and S13), $\overline{Z}_{min}$ (SI Figures S14 and S15), $\overline{Z}_{ave}$ (SI Figures S16 and S17) and $\overline{Z}_{mid}$ (Figure 5(A) and SI Figure S18) reveal that ensembles generated with bmBFs have lower values of all four $Z$-parameters compared to the ensemble generated with EFs. This is consistent across all $10$ reconstructed GRNs. Restricting to the ensembles generated with bmBFs, we find that $\overline{Z}_{max}$ and $\overline{Z}_{ave}$ do not show any particular trend across the $10$ GRNs, whereas $\overline{Z}_{min}$ and $\overline{Z}_{mid}$ consistently achieve the lowest values in the NCF-ensemble, across all $10$ GRNs. Note that the $\overline{Z}_{min}$ for the NCF-ensembles always have zero variance since $Z_{min}^{NCF}$ depends only on $k$ (Equation (4) and SI text, section 9 for a proof).

The distributions of our last descriptor of local dynamics, network sensitivity denoted by $\overline{s}$, are shown in Figure 5(B) and SI Figure S19, indicating that NCFs lead to the lowest values compared to all other BFs (in agreement with previous observations [19]).

Lastly, the Spearman correlation coefficient among the five descriptors of local dynamics, as given via heatmaps in SI Figures S20 and S21, show that most pairs of descriptors show moderate to very strong positive correlation across the various GRNs. This is illustrated in particular via scatter plots between $\overline{Z}_{ave}$ and $\overline{Z}_{mid}$ (SI Figures S22 and S23), and $\overline{s}$ and $\overline{Z}_{mid}$ (SI Figures S24 and S25). In the following section, we explore which of these descriptors can serve as good proxies of bushiness or convergence.

### Descriptors of local dynamics can serve as good proxies of bushiness of STGs

Wuensche showed that, in CA, the $Z$-parameter exhibits a ‘marked’ correlation with the *G*-density [45], suggesting that it may be possible to infer at least qualitative information about the bushiness of the STG of a CA from properties of its rule table. Extending this question to the BN framework, we ask whether descriptors of local dynamics (in fact, characteristics of the rule tables for each node) can serve as good proxies of bushiness of such a model’s STG (Figure 2).

**Figure 6:**
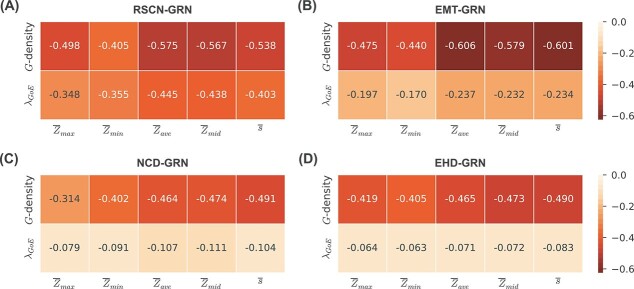
**Correlation heat map between descriptors of local dynamics and global measures (bushiness and convergence rate) for ensembles generated using network structures from four published Boolean GRNs.** The EF-ensemble for each network structure was used to generate the heat maps. The rows of each subplot correspond to quantities computed on the STG, namely *G*-density and $\lambda _{GoE}$. The columns of each subplot correspond to descriptors of local dynamics, namely, the network $Z$-parameters ($\overline{Z}_{max}$, $\overline{Z}_{min}$, $\overline{Z}_{ave}$, $\overline{Z}_{mid}$) and network sensitivity ($\overline{s}$). The heat maps show the pair-wise Spearman correlations between descriptors of local dynamics and global measures of bushiness and convergence rate defined on the state transition graph. The descriptors of local dynamics show a moderate to strong negative correlations with *G*-density and very weak to moderate negative correlations with $\lambda _{GoE}$.

To do so, we compute the Spearman correlation coefficients of the *G*-density with the different descriptors of local dynamics as given in the heatmaps in Figure 6 and SI Figure S26. From these figures, it is clear that for most GRNs, $\overline{Z}_{ave}$, $\overline{Z}_{mid}$ and $\overline{s}$ show a moderate to strong negative correlation with the *G*-density and are more strongly correlated with it than are $\overline{Z}_{max}$ and $\overline{Z}_{min}$. This suggests that those three descriptors may serve as useful proxies of the bushiness. Note that since $\langle d_{in} \rangle _{non-GoE}$ is a monotone increasing function of the *G*-density (see Equation (1)), the Spearman correlation coefficients of $\langle d_{in} \rangle _{non-GoE}$ with the five descriptors will be the same as that of *G*-density and hence are not shown in the heat maps. The scatter plots of *G*-density with each of the five descriptors of local dynamics are provided in the SI: $\overline{Z}_{max}$ (SI Figures S27 and S28), $\overline{Z}_{min}$ (SI Figures S29 and S30), $\overline{Z}_{ave}$ (SI Figures S31 and S32), $\overline{Z}_{mid}$ (SI Figures S33 and S34) and $\overline{s}$ (SI Figures S35 and S36).

An implication of these correlations is that for ensembles of larger networks (where generating the STG is computationally infeasible), we may simply use the descriptors of local dynamics such as $\overline{Z}_{mid}$ and $\overline{s}$ to predict which models will have a more bushy STG. For instance, if one selects the models having average relative rank of $0.05$ for $\overline{Z}_{mid}$, the corresponding average relative rank for the *G*-density is typically $0.2$, very far from the value of $0.5$ which would arise if $\overline{Z}_{mid}$ had no predictive power (SI Figures S37 and S38). To assess more globally the relevance of using $\overline{Z}_{mid}$ as a proxy for *G*-density, we computed the corresponding AUROC and AUPRC measures (see SI text, section 11 and SI Table S42). From the table, we observe, for instance, that the average AUROC value consistently exceeds $0.72$ for majority of the GRNs, indicating favorable performance in this context.

### The average convergence rate of trajectories originating at random states is a good proxy for the average convergence rate of trajectories originating at *GoE* states

Analogous to the case of *G*-density, we compute the correlation of $\lambda _{GoE}$ with the descriptors of local dynamics (Figure 6 and SI Figure S26) and find that they are negative as expected, but quite weak for most ensembles. Therefore, it is inappropriate to use any of these descriptors as proxies for $\lambda _{GoE}$. Note, nevertheless, that for most GRNs, the $\overline{Z}_{ave}$, $\overline{Z}_{mid}$ and $\overline{s}$ show stronger correlations than $\overline{Z}_{max}$ and $\overline{Z}_{min}$. The scatter plots between the $\lambda _{GoE}$ and each of the five descriptors of local dynamics are provided in the SI: $\overline{Z}_{max}$ (SI Figures S39 and S40), $\overline{Z}_{min}$ (SI Figures S41 and S42), $\overline{Z}_{ave}$ (SI Figures S43 and S44), $\overline{Z}_{mid}$ (SI Figures S45 and S46) and $\overline{s}$ (SI Figures S47 and S48).

As an alternative to these descriptors, we propose $\lambda _{random}$ (see Methods) as a possible proxy for $\lambda _{GoE}$. Our grounds for this proposition is that $\lambda _{GoE}$ is very strongly correlated with $\lambda _{all}$ (SI Figures S49 and S50), which provides an impetus to compute $\lambda _{random}$ because, if so, one no longer has to compute the STG. SI Figures S51 and S52 reveal that $\lambda _{random}$ can indeed serve as an excellent predictor of $\lambda _{GoE}$.

## DISCUSSION AND CONCLUSIONS

In the context of CA, Wuensche showed that the signature of highly ordered or convergent dynamics is the presence of short, highly branching transient trees, with a high proportion of leaves in the STG, whereas the signature of chaotic dynamics is long transients, low branching in the STG and a low proportion of leaves therein [45, 47, 54]. Such characterizations are absent in generalized automata that mimic biological systems, namely, BNs—which generally lack a regular network architecture and employ heterogeneous rules across their nodes. This work is a first such study of the bushiness and convergence of STGs for different reconstructed GRNs using *G*-density and $\lambda _{GoE}$, leading to the conclusion that bmBFs lead to more bushy and convergent STGs compared to random EFs. As a remark, the use of 10 different GRNs makes our results very general and minimizes any bias that may be associated with particular GRNs. Note, bmBFs occupy a tiny fraction in the space of all BFs and consequently can severely restrict the space of biologically plausible Boolean models (SI Tables S32–S41) and enable model selection therein [51].

Contrary to our expectation that higher bushiness (i.e., higher contraction rate) results in higher convergence, we were surprised to find that the *G*-density and $\lambda _{GoE}$ measures are only weakly correlated. A posteriori, it is easy to convince oneself that the two measures indeed capture different features. For example, consider a rooted tree of constant connectivity $K$ for which all the leaves are at the same distance $H$ from the root. The bushiness, that is *G*-density, is close to $(K-1)/K$, while the transient times are all equal to $H$, leading to a convergence rate of $1/H$. Thus, bushiness and convergence rate can be varied essentially independently.

In our study we were able to exhaustively enumerate the *GoE* states of all the models. Unfortunately, the computational complexity of identifying the *GoE* states in BNs is a #$\mathbf{P}$-complete problem, meaning that it is at least as hard as a NP-complete problem to compute the *G*-density of a general BN [53]. Thus, it is necessary to introduce proxies that can bypass this computational complexity to give us information about the bushiness of the STG without having to find its *GoE* states. Among all the network $Z$-parameters we proposed and explored, the choice $\overline{Z}_{mid}$ typically showed the highest correlation with *G*-density and for the class of NCFs it is highly scalable. We found that the network sensitivity ($\overline{s}$) is also a good proxy of *G*-density. This is justified by the fact that NCFs, known to produce more ordered dynamics [19, 22] compared to EUFs and EFs, achieve the minimum average sensitivity over all possible BFs [19], thereby leading to lower values of $\overline{s}$. Such dynamics are directly linked to the robustness of the system to perturbations, and, therefore, a highly bushy STG is a signature for very robust dynamics. Note that the descriptors defined at the level of individual nodes are ‘unaware’ of the network architecture (which is not an issue in CA). $Z$-parameters other than the four discussed in this work may be defined of course. For instance, one could consider the median of all $Z_{left}$ values of a BF. But we find that this measure is more complicated to compute compared to $Z_{mid}$, and when computed at the network level, correlates more weakly with *G*-density compared to $\overline{Z}_{mid}$ or $\overline{Z}_{ave}$. Since the average convergence rate correlates only very weakly with the descriptors of local dynamics, we tested and found that the average convergence rate of the trajectories of randomly sampled states from the STG ($\lambda _{random}$) serves as an excellent proxy for $\lambda _{GoE}$. In sum, we provide three proxies: $\overline{Z}_{mid}$ and $\overline{s}$, which inform us about the ‘bushiness’ or contraction rate of phase space arising under BN dynamics, and $\lambda _{random}$, which informs us about the ‘convergence’ rate of those dynamics.

Notwithstanding the developments of new methods and results, there are some limitations that arise in our work that we would like to address in the future. (i) To propose algorithms that can sample EUFs more uniformly than the one presented in this work. Although our current sampling bias is small and does not affect our conclusions (most of the networks studied have nodes with at most six inputs), it certainly will strengthen the conclusions for networks having nodes with more than six inputs. (ii) To devise algorithms to speed up the computation of $Z$-parameters, since it is computationally expensive to obtain the $Z$-parameter for nodes with more than six inputs because of the $k!$ permutations of the inputs that have to be generated. (iii) To extend the computation of global measures to STGs generated under asynchronous update. It can be expected that the number of *GoE* states under asynchronous update is almost always less than what is obtained in the synchronous case. Can one define a *G*-density that is computationally tractable in that situation? Of course this question also applies to our measure $\lambda _{GoE}$. (iv) Finally, to search for proxies of $\lambda _{GoE}$ solely at the level of the BFs.

In conclusion, we have systematically characterized how global BN robustness measures, namely rate of contraction and of convergence, vary depending on the type of logic rule employed by drawing inspiration from the theory of CA, which likely can provide further insights into understanding dynamics of BNs. We also introduced a novel tool $\overline{Z}_{mid}$, which is inspired from CA, to the Boolean network community alongside existing measures aimed at predicting robustness of these dynamics.

**Key Points**

- We quantify structural features of state transition graphs (STGs) of Boolean networks using measures based on their garden-of-Eden states.
- We adapt the Z-parameter, defined on rules in elementary cellular automata, to Boolean functions in Boolean networks.
- Biologically meaningful Boolean functions lead to highly bushy and convergent STGs.
- The Z-parameter and the average sensitivity can serve as good proxies for the bushiness of the STG.
- The average convergence rate of trajectories originating at random states serves as an excellent proxy for the case of trajectories originating at garden-of-Eden states.

## Supplementary Material

SI_BushySTG_R2_bbae150

## Data Availability

All data and codes needed to reproduce the results in this manuscript are deposited in GitHub and are available at: https://github.com/asamallab/BushySTG.

## PREPRINTS

A preprint of this article has previously appeared at: https://doi.org/10.1101/2023.07.17.549398.

## Author Biographies

**Priyotosh Sil** is a PhD student in the Computational Biology group at The Institute of Mathematical Sciences (IMSc), a CI of Homi Bhabha National Institute (HBNI), Chennai, India. He is interested in mathematical modeling of biological systems.

**Ajay Subbaroyan** is a PhD student in the Computational Biology group at IMSc, Chennai, India. He is interested in modeling biological systems using computational and mathematical approaches.

**Saumitra Kulkarni** is a project assistant in the Computational Biology group at IMSc, Chennai, India. He is interested in Network Science and its applications in biological, social and financial systems.

**Olivier C. Martin** is an INRAE Research Director at the Institute of Plant Sciences, University Paris-Saclay, France. He uses computation and statistics to understand the workings of biological systems.

**Areejit Samal** is a Professor in the Computational Biology group at IMSc, Chennai, India. He is interested in applications of network science to complex biological systems.
